# Genetic evaluation of Addison's disease in the Portuguese Water Dog

**DOI:** 10.1186/1746-6148-2-15

**Published:** 2006-05-02

**Authors:** AM Oberbauer, JS Bell, JM Belanger, TR Famula

**Affiliations:** 1 Department of Animal Science, University of California, Davis, California, USA; 2 Department of Clinical Sciences, Tufts Cummings School of Veterinary Medicine, North Grafton, Massachusetts, USA

## Abstract

**Background:**

Addison's disease, also known as hypoadrenocorticism, has been reported in many individual dogs, although some breeds exhibit a greater incidence than the population as a whole. Addison's is presumed to be an autoimmune mediated hereditary defect but the mode of inheritance remains unclear. In particular, the heritability and mode of inheritance have not been defined for the Portuguese Water Dog although Addison's is known to be prevalent in the breed.

**Results:**

The analyses present clear evidence that establishes Addison's disease as an inherited disorder in the Portuguese Water Dog with an estimate of heritability of 0.49 (± 0.16); there were no differences in risk for disease across sexes (p > 0.49). Further, the complex segregation analysis provides suggestive evidence that Addison's disease in the Portuguese Water Dog is inherited under the control of a single, autosomal recessive locus.

**Conclusion:**

The high heritability and mode of inheritance of Addison's disease in the Portuguese Water Dog should enable the detection of segregating markers in a genome-wide scan and the identification of a locus linked to Addison's. Though the confirmation of Addison's disease as an autosomal recessive disorder must wait until the gene is identified, breeders of these dogs may wish to keep the present findings in mind as they plan their breeding programs to select against producing affected dogs.

## Background

Addison's disease, also referred to as chronic adrenal insufficiency or hypoadrenocorticism, has been reported in many individual dogs. Although mixed breeds present as the "breed" most frequently diagnosed with Addison's, certain pure-breds, including the Great Dane, Rottweiler, Standard Poodle, Portuguese Water Dog, West Highland White Terrier, and Wheaton Terrier, experience a disproportionate level of prevalence of the disease [1,2]. The defining characteristic in Addison's disease is the failure of the adrenal cortex to produce sufficient quantities of steroid hormones. Addison's can be characterized as either primary or secondary depending upon the site of impairment, with primary being most common in dogs [2]. The symptoms associated with Addison's are often diffuse reflecting the general metabolic influence of the hormones produced by the adrenal cortex. Symptoms include lethargy, vomiting, inappetence, depression, and weight loss, [2-4].

Addison's disease in humans has long been characterized as an inherited trait [5,6] induced by autoimmune-mediated destruction of the adrenal cortex. Similar to the human, the majority of dogs present with primary Addison's disease in which the adrenal cortex exhibits severe deterioration mediated by an autoimmune reaction [7]. An inherited susceptibility for Addison's has been proposed for more than 30 dog breeds [8]. Yet even though canine Addison's is well documented to run in families [9-12], the mode of inheritance is unclear. One breed, the Standard Poodle, exhibits the presence of a single major autosomal locus affecting expression [10], while another breed, the Bearded Collie, has a less definitive mode of inheritance for Addison's [11]. We undertook genetic analysis to characterize the inheritance of Addison's in a third breed having a low genetic relationship to the Standard Poodle [13] to clarify whether unrelated breeds demonstrate unique modes of inheritance for Addison's as has been demonstrated for other disorders such as blood clotting [14-16]. In particular, the heritability and mode of inheritance have not been defined for the Portuguese Water Dog although breeders recognize that Addison's is a concern in the breed (Canine Health Foundation, American Kennel Club 2001 survey). Knowledge of a genetic influence on the expression of Addison's in the Portuguese Water Dog would assist breeders in making breeding decisions that would decrease the incidence of Addison's in their breed. In this study, the heritability and the mode of inheritance, as determined by complex segregation analysis, are reported for the Portuguese Water Dog.

## Results

### Heritability

Table 1 presents evidence that establishes Addison's disease as an inherited disorder in the Portuguese Water Dog. The narrow sense estimate of heritability of 0.49 (± 0.16) supports a significant inherited component for this illness, though a difference in risk for disease across sexes was not supported (p > 0.49).

### Complex segregation analysis

Table 2, presenting the results of the complex segregation analysis, provides a clearer picture of the inherited nature of this disorder. The results presented in Table 2 establish that the most plausible genetic explanation for this disease is the action of a single recessive locus. Note, for all the parameters, the 95% highest density region (HDR) does not include zero, a clear demonstration of the presence of a major locus for Addison's disease in the Portuguese Water Dog. Not presented was the equivalent analysis accommodating non-Mendelian transmission of the putative major allele. Such an analysis, when evaluated through the 95% HDR, did show an overlap with the Mendelian transmission estimates demonstrating that the Mendelian model provides the best fit to the data, one of the criteria established by Elston [17].

Of the total dogs in this study, 590 dogs were inbred; their average inbreeding coefficient was 0.061. There were as many as seven overlapping generations included in the families under study and not all dogs within the study had equivalent number of generations evaluated. Nevertheless, with an eye toward making simple recommendations to breeders, the relationship between inbreeding and the risk for Addison's disease should be evaluated. While the data set does not permit computation of the level of inbreeding of Portuguese Water Dogs as a whole, the relationship between Addison's and inbreeding can be assessed. Classifying affected and unaffected dogs by their inbreeding coefficient can reveal a simple relationship between level of inbreeding and the presence of disease. In the present study, among dogs with an inbreeding coefficient between zero (0.0) and 0.05, 11 of 205 dogs (5.4%) were recorded as affected by Addison's. However, as the level of inbreeding increased, the risk of Addison's disease increased with it. Specifically, for Portuguese Water Dogs with an inbreeding coefficient between 0.05 and 0.10 there were 8 affected dogs among a total of 97 dogs (8.3%). For those dogs with an inbreeding coefficient between 0.10 and 0.15 there were 15 of 70 (21.4%) dogs affected by Addison's. Though a small sample, among dogs with an inbreeding coefficient greater than 0.15 there were 5 affected dogs among a total of 23 (21.7%). This result is consistent with the results of Table 2 in that inbreeding increases homozygosity, and a disease that is strongly influenced by a segregating recessive locus should see an increased incidence among a cluster of inbred animals.

## Discussion

The objective of this study was to assess the genetic contribution to the expression of Addison's disease in the Portuguese Water Dog. The results provide sufficient and convincing evidence that Addison's disease in the Portuguese Water Dog is an inherited disorder. The mode of inheritance, determined in the present data set, is consistent with a single, autosomal recessive locus. This finding should be helpful to breeders as they struggle to reduce the prevalence of this disorder in the breed. Unfortunately, given the relatively late onset of illness, the evaluation of dogs at risk for expressing Addison's disease, or identification of carriers, is not straightforward. However, the evidence for the contributions of a single locus (Table 2) suggests that a genome scan for the putative disease-causing allele is advisable. Identification of the allelic mutation would enable the identification of dogs at risk or carriers prior to their contribution to the gene pool.

**Figure 1:**
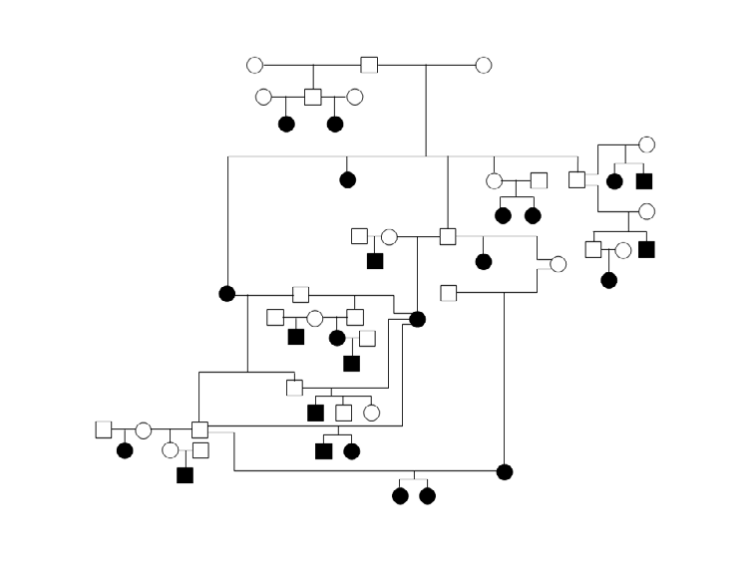
**Subset pedigree of Portuguese Water Dogs with heritable Addison's disease**. All dogs in this subset trace back to common ancestors not depicted in this figure. Male dogs are represented by squares and female dogs, circles. Filled in symbols represent confirmed Addisonian dogs.

Comparison of Tables 1 and 2 also demonstrates a basic equivalence in estimates of the narrow sense heritability across the two computational approaches. That is, the complex segregation analysis also provides an estimate of a polygenic contribution to disease. The estimate of the mean of the posterior density for the polygenic variance component was 1.08, implying an estimate of heritability of the polygenic component as 0.52 = 1.08/(1.08 + 1.00); a value well within the margins of error for the heritability estimate in Table 1. Note that the 95% HDR for the polygenic variance does not include 0.0 but the interval does indicate that the polygenic contribution could be very small. This suggests that the putative major locus for Addison's disease is not likely to be the sole determinant of the disease expression. The genetic contributions of the polygenic component may reflect other genes influencing the progression of the disorder. Alternatively, inclusion of dogs as "unaffected" in the statistical analyses when in fact the dogs have not yet expressed the disease may influence our estimate of the posterior density of the polygenic variance. This in turn may disturb our estimates of the limits for the 95% HDR affecting our conclusions on the potential for a polygenic contribution to Addison's disease over and above that of the putative major locus. An additional caution must be exercised concerning dogs that are designated Addisonian when in fact they may not be due to iatrogenic adrenal suppression [18] or other instances of adrenal insufficiency. Accurate diagnosis of the disorder is critical to any genetic transmission study due to artificially inflated statistical significance if inaccurately diagnosed dogs are incorrectly classified. While these uncertainties remain, in the present study, the consistency of transmission of Addison's disease (Figure 1) and the reliance upon veterinarian diagnoses should mitigate those concerns.

Though not presented in Table 2 for the sake of brevity, several models were fit in the complex segregation analysis. Specifically, fitting these models are intended to reduce the probability of a false detection of a major locus [17]; models in which the assumption of Mendelian transmission of the alleles of the putative major locus are relaxed. The null model is Mendelian transmission, and this model should be a significant improvement for the likelihood over the no-major locus model if we are to draw the conclusion that a major locus is segregating in this population of dogs. "Improvement", when working in the Bayesian framework provided by iBay [19], is reliant upon the examination of the 95% HDR. In our examination of Addison's disease in the Portuguese Water dog, allowing for non-Mendelian transmission of the putative major alleles did not improve the fit: the 95% HDR for the transmission probabilities demonstrated considerable overlap with the Mendelian transmission values. For this reason we accept the conclusion of the simplest null model that of the major locus model with alleles moving from parent to offspring with the typically expected probabilities.

Though complex segregation analysis is fraught with challenging statistical issues, the estimate of the allele frequency for the putative disease allele in the base population is 0.49 (i.e., frequency of allele "A" = .51, with frequency of the disease-causing "B" allele = 1. – .51 = .49; see Table 2). Accordingly, for our suggested major locus model one would predict that 24% of dogs would be affected with Addison's disease in a randomly mating population. The observed incidence in the data set is considerably lower with 103 affected dogs out of 804 (103/804 = 0.128), suggesting that breeders may be selecting parents so as to reduce the risk of disease. Such a high expected prevalence of Addison's greatly increases the power of identifying linkage by facilitating the detection of segregating markers in a genome-wide scan. In an effort to simply assess whether the Portuguese Water Dog family in the present study offers the power to detect genetic linkage with the Addison's phenotype, we simulated a recessive locus perfectly linked with Addison's in a subset of the larger Portuguese Water Dog family and computed expected LOD scores in a linkage analysis. The predicted LOD score of a marker within 5 cM of the Addisonian locus in a small subset family of 142 dogs was 16.55 using the CRIMAP analysis program [20] giving support to the concept that the family under study offers the power to detect genetic linkage with the Addison's phenotype.

As in a similar analysis of the Standard Poodle [10], there is no evidence for a sex difference in risk of Addison's disease for Portuguese Water Dogs. This suggests that the literature reports of a greater prevalence among females may represent an artifact of the sample populations evaluated: dogs referred to teaching hospitals rather than dogs evaluated by all veterinarians [1,12]. The results of the present study also are consistent with the published evidence of the mode of inheritance for Addison's disease in the Standard Poodle [10]. In both the Portuguese Water Dog and the Standard Poodle, the data support the view that the disease is inherited as a single autosomal recessive trait. In contrast, although highly heritable, the mode of inheritance of Addison's disease in the Bearded Collie is less clear [11]. This disparity in the mode of inheritance predictions possibly stems from the variability in the onset of the disorder and the classification of dogs as "unaffected" in the statistical analyses when in fact the dogs have not yet expressed the disease. The consistency of high heritability for the Addisonian phenotype across these different breeds supports the possibility that Addison's may have a common mode of inheritance for dogs in general. This concept is supported given the high incidence among mixed breeds [2] and the recent determination that large haplotype regions are shared across multiple dog breeds suggesting that genetic liability predisposition may likewise be shared [21].

## Conclusion

Until early clinical detection of Addisonian status exists or genetic tests are available to identify dogs at risk for developing Addison's disease or those dogs that are carriers of the mutant allele, breeders of Portuguese Water Dogs should consider the genetic contribution to the expression of Addison's disease. Although the present study cannot confirm the mode of inheritance, the putative mode of inheritance as an autosomal recessive disorder suggests that breeders could actively select against generating affected dogs in their breeding programs.

## Methods

### Animals

Portuguese Water Dogs have been noted to have a disproportionate number of Addisonian cases [1,2]. To clarify the heritability and predict the mode of inheritance of Addison's disease in the Portuguese Water Dog, a study was initiated in conjunction with the Portuguese Water Dog Club of America. Interested owners of Portuguese Water Dogs submitted survey questionnaires which asked for registered name, sire and dam, date of birth, sex, whether intact or altered, whether Addisonian or not, how diagnosis was made, age of diagnosis, and whether the dog received steroid treatment prior to diagnosis. Pedigree information was obtained from the American Kennel Club records.

A dog was designated as Addisonian if the diagnosis was made following an adrenocorticotropin hormone (ACTH) stimulation test administered by a veterinarian. The ACTH test measures the competence of the adrenal cortex by evaluating blood cortisol concentrations before and after the exogenous ACTH stimulation. In a clinically normal animal, the baseline blood cortisol prior to the ACTH stimulation would be 0.5 to 4.0 μg of cortisol/dl that would rise to 8.0 to 20 μg/dl following ACTH stimulation. Dogs failing to respond to the ACTH stimulation with elevated circulating cortisol are classified as Addisonian [22]. Most owners were prompted to test their Portuguese Water Dogs for Addison's disease because of signs of lethargy, vomiting, or collapse. The ACTH stimulation criteria for diagnosis allows for an accurate and repeatable assessment of the disease. Dogs included in the present study were classified as either Addison-affected (based on the above criteria) or unaffected Portuguese Water Dogs.

### The data

Addisonian disease incidence data were collected on 804 Portuguese Water Dogs from the United States, though with pedigree information an additional 1,273 animals were included in the study; these additional animals did not have a recorded observation for Addison's disease. The total of 2,077 dogs included in the analysis is derived from one large family (with 2,051 dogs), three families of seven dogs, and 5 dogs of unassigned pedigree. The only additional variable included in our analysis was sex. No additional phenotypic information was recorded along with the diagnosis of Addison's disease (e.g., coat color). Specifically, there were 458 females (396 unaffected and 62 affected) and 346 males (305 unaffected and 41 affected) with known health status. Figure 1, representing a small subset of the dogs in the study, is provided to demonstrate transmission of the disorder. However, the parents of all affected Portuguese Water Dogs in this study trace back to common ancestors. The Portuguese Water Dog population in the United States was established from two separate Portuguese kennel lines (Algarbiorum and Alvalade) beginning in the late 1960s [23].

For the affected dogs included in the analyses, the mean age of Addisonian diagnosis was 46 months (median 36 months). The mean age of the dogs in the study designated unaffected was 48.5 months (median 37 months). Caution must be exercised to avoid designating a dog as being Addisonian when in fact the responsiveness of the adrenal has been blunted by exogenous administration of glucocorticoids. For 6.4% of the Addisonian dogs in the present study, owners reported that the dogs had received some sort of short term steroid treatment at some point prior to the Addisonian diagnosis. However, in the United States, the standard veterinary protocol for an ACTH stimulation test is to gradually withdraw exogenous corticosteroids prior to administration of the ACTH challenge (R. Nelson, personal communication). In addition, those survey questionnaires that included the ACTH stimulation test values, Addisonian dogs had pre and post-ACTH cortisol levels of less than 0.2 μg/dl which differ from the expected values of a clinically normal dog as stated earlier. Taken together, this suggests that the diagnosis of Addison's disease for the dogs in this study were Addisonian and not phenocopies. For the dogs with alteration status recorded, the vast majority of dogs (88%) were diagnosed after being reproductively altered.

### Estimation of heritability

For the objective of estimating the heritability of Addison's disease in the Portuguese Water Dog, a threshold model for the liability to disease was used. This method assumes that a dog can be assigned to a specific disease class (unaffected/affected) when an underlying, unobservable risk (or liability) for disease exceeds a threshold of τ = 0. The distribution of the unobservable liability was assumed to be multivariate normal. The correlation in liability of two dogs *i *and *j *was modeled to be ρ_ij _= a_ij _h^2 ^+ δ_ij _σ_e_^2 ^where ρ_ij _is the correlation in liability to disease between dogs *i *and *j*; a_ij _is the additive relationship between dogs *i *and *j*; h^2 ^is the narrow sense heritability of liability to disease; δ_ij _is the coefficient for the random environmental component for dogs *i *and *j *such that δ_ij _equals 1 if *i *equals *j *and zero otherwise; and σ_e_^2 ^= 1 - h^2^, with no loss of generality. The null hypothesis of no genetic contribution (i.e., h^2 ^= 0) was tested with a likelihood ratio test, comparing the full model likelihood with that of the restricted model where h^2 ^= 0. Heritability is expressed as the mean ± standard error of the mean. It should be noted that because the data represent owner submissions, the data were collected in a non-random fashion. Further, being a study of inheritance, the data set was constructed around probands. Such data require an adjustment for ascertainment bias. However, the mixed linear models utilized in this study accommodate nonrandomly sampled data [24] as long as the dogs added into the study to complete the pedigree associations can be considered a random sample of Portuguese Water Dogs. In addition, a test of the effect of sex on the liability of Addison's disease was also tested through the likelihood ratio test. Calculations were implemented through the computer program SOLAR [25,26], making use of the binary trait analysis first described in Duggirala *et al*. [27].

### Complex segregation analysis

The possibility that Addison's disease in Portuguese Water dogs is influenced by the action of a single segregating locus of large effect can also be examined. Complex segregation analysis, developed by Bonney [28], is intended to integrate Mendelian transmission genetics at a single locus with the patterns of covariance expected in polygenic inheritance. Lynch and Walsh [29] provide a more complete description of complex segregation analysis. Elston *et al*. [17] outlined the criteria that must be satisfied before acceptance of the single major locus model so as to reduce the risk of false positive declarations of a major locus model. Evaluation of the models necessary for complex segregation analysis was conducted with the Bayesian software package iBay [19]. The iBay software is an extension of MaGGic [30], rewritten to accommodate complex segregation analysis in binary traits for pedigrees that include inbreeding.

The software selected to conduct the complex segregation analysis is built upon a Bayesian foundation, making use of Monte Carlo Markov chains (MCMC) through a Gibbs sampler. Accordingly, point estimates of unknown parameters are not derived, but rather estimates of the posterior density for unknown parameters. The iBay [19] package was recently used to evaluate the contribution of a major locus to osteochodral diseases in pigs [31], where a more complete outline of the MCMC approach is detailed. The goal of this strategy was to simultaneously estimate the posterior density for a polygenic contribution to Addison's disease along with the contributions of a putative Mendelian locus. Specifically, for this mixed-inheritance model, the strategy allowed the evaluation of a polygenic variance component, the additive and dominance contributions of a single locus (the parameters -a, d, and a for the putative major locus genotypes AA, AB, and BB, respectively) and the frequency of allele A of the putative major locus (defined as *"q"*). Given our scoring of phenotypes, where affected is 1 and unaffected scored as zero, the "B" allele represent the putative disease-enhancing allele. Note also that the iBay software models the unobservable scale of this threshold trait such that the residual variance is fixed at 1.0 (i.e., σ_e_^2 ^= 1).

Creation of the Gibbs sample requires several key assumptions about the behavior of these unknown parameters. Though a variety of models can be considered, all are some variant of the following: sex as a fixed effect with a flat (i.e., uniform) prior density, the polygenic variance component with a flat prior density, as well as flat prior densities for the additive, dominance, and allele frequency parameters. A Gibbs sample of 9,000 was generated, beginning with the creation of 350,000 total samples, a "burn-in" of 50,000 and a sampling rate of every 100-th Gibbs value. This process was repeated two additional times, to create three replicate chains. From the 9,000 Gibbs samples, the mean, standard deviation, mode and the upper and lower limits of a 95% highest density region (HDR) was computed for each of the unknown parameters. HDR were computed according to Hyndman [32] with the public domain software hdrcde [33], a package within the R-program [34].

## Authors' contributions

AMO and JSB jointly conceived of the study with input on design by TRF. JMB and AMO coordinated the study. TRF carried out the statistical analyses. AMO and TRF drafted the manuscript with editing by JSB and JMB. All authors read and approved the final manuscript.

## Figures and Tables

**Table 1:** Estimate of heritability and gender contrast in a threshold model for Addison's disease.

	Estimate	Standard Error	P-Value
	
Heritability	0.49	0.16	<0.01
Females – Males	-0.09	0.13	0.49

**Table 2:** Marginal posterior means, modes, standard deviations and limits to the 95% highest density regions (HDR) of model parameters for Addison's disease in Portuguese Water dogs in a Bayesian mixed-inheritance model with a completely recessive major locus.

	Polygenic Variance	Major Locus Variance	Additive Effect (a)	Dominance Deviation (d)	Frequency (q)
Mean	1.08	6.20	2.82	-2.82	0.51
Mode	0.79	5.24	2.68	-2.68	0.52
SD	0.52	1.81	0.43	0.43	0.06
HDR 95% Low	0.14	3.19	2.02	-3.63	0.40
HDR 95% High	1.96	9.72	3.63	-2.02	0.63
